# One-Pot Synthesis of Nanostructured Ni@Ni(OH)_2_ and Co-Doped Ni@Ni(OH)_2_ via Chemical Reduction Method for Supercapacitor Applications

**DOI:** 10.3390/ma16010380

**Published:** 2022-12-30

**Authors:** Seungyong Eom, Jinjoo Jung, Do Hyung Kim

**Affiliations:** Nano Applied Physics Laboratory, Department of Physics, Kyungpook National University, Daegu 702–701, Republic of Korea

**Keywords:** core–shell, nickel hydroxide, chemical reduction, formation mechanism, hydrothermal, supercapacitor

## Abstract

Crystalline Ni@Ni(OH)_2_ (cNNH) and Co-doped cNNH were obtained via a simple one-pot hydrothermal synthesis using a modified chemical reduction method. The effect of each reagent on the synthesis of the nanostructures was investigated concerning the presence or absence of each reagent. The detailed morphology shows that both nanostructures consist of a Ni core and Ni(OH)_2_ shell layer (~5 nm). Co-doping influences the morphology and suppresses the particle agglomeration of cNNH. Co-doped cNNH showed a specific capacitance of 1238 F g^−1^ at 1 A g^−1^ and a capacitance retention of 76%, which are significantly higher than those of cNNH. The enhanced performance of the co-doped cNNH is attributed to the reduced path length of the electrons caused by the decrease in the size of the nanostructure and the increased conductivity due to Co ions substituting Ni ions. The reported synthesis method and electrochemical behaviors of cNNH and Co-doped cNNH affirm their potential as electrochemically active materials for supercapacitor applications.

## 1. Introduction

Supercapacitors are attractive energy storage devices with the advantages of high power density, long cycle life, and fast charge–discharge cycling [1,2]. The electrochemical performance of supercapacitors depends primarily on the active material and the architecture of the working electrode. The pseudocapacitive materials such as transition metal oxides and conductive polymer have been used as active materials [3]. Among the many functional materials available, nickel hydroxide (Ni(OH)_2_) is promising for use in supercapacitors because of its high theoretical specific capacitance, well-defined redox behavior, and low cost [4]. As is well known, Ni(OH)_2_ can be crystalline with two polymorphs, α and β, or amorphous with a disordered structure. When used as an active material for supercapacitors, α-Ni(OH)_2_ outperforms β-Ni(OH)_2_ because the former is composed of a two-dimensional layered structure with abundant intercalated anions, resulting in high ion conductivity and electrochemical activity [5,6,7]. However, in an alkaline environment, the anions in the interlayers in α-Ni(OH)_2_ easily escape, and consequently, it is readily converted into β-Ni(OH)_2_ [8].

Amorphous or low-crystalline Ni(OH)_2_ has the potential to be used as an active material for supercapacitors owing to numerous active sites and isotropic ion diffusion paths within its disordered structure compared with crystalline Ni(OH)_2_ [9]. However, low electrical conductivity limits the redox reaction inside the material and leads to an ohmic drop, turning the rich active sites inside the material into dead volumes [10]. Therefore, to improve the performance of amorphous or low-crystalline Ni(OH)_2_-based supercapacitors, the issues of low electrical conductivity and the short cycle life of these materials should be addressed.

One approach to overcome this problem is to construct core–shell nanostructures that employ highly conductive metallic cores with electroactive shells, such as Ni@Ni(OH)_2_ structures [11]. Amorphous or low-crystalline Ni(OH)_2_, deposited or assembled on highly conductive materials, such as carbon materials and transition metals, encompasses an efficient architecture to enhance the electrical conductivity [12,13]. Further, the architecture composed of nanostructures satisfies the requirements for increasing the specific surface area while decreasing the diffusion path of the protons. However, the construction of these architectures is a complicated process involving several steps of deposition or template removal. 

Another approach to improve the performance is the ionic substitution of other elements. The use of substitutes such as Al, Ca, Zn, Y, and Co is effective in improving the performance of the electrode. In particular, Co ions are attractive because they increase the number of proton vacancies and the diffusion coefficients of protons and utilize the active material at a high charge–discharge rate [14]. In addition, because the radius of the Co ion is similar to that of the Ni ion, it could easily substitute the Ni ion without severe lattice strain [15]. Therefore, doping with Co ions improves the conductivity and stability of Ni(OH)_2_. 

In this study, we developed and analyzed an efficient method involving a simple hydrothermal reaction for the one-pot synthesis of core–shell structured crystalline Ni@Ni(OH)_2_ (cNNH). We employed a chemical reduction method, wherein N_2_H_4_·H_2_O was used as the reducing reagent, NaOH was used as the alkaline reagent, and poly(vinyl pyrrolidone) (PVP) acted as the surfactant. Based on the investigation of the role of the reagents, a mechanism for the synthesis of cNNH is proposed. The effects of Co-doping on cNNH and the electrochromic performance of cNNH and Co-doped cNNH were investigated. The Co ion partially substituted the Ni ions, resulting in an increase in the concentration of holes and the specific surface area, thereby reducing the charge-transfer resistance and increasing the accessibility of electrolyte, which are useful for enhancing the performance of supercapacitors.

## 2. Materials and Methods

High-purity nickel(II) chloride hexahydrate (NiCl_2_·6H_2_O, 99%), cobalt(II) chloride hexahydrate (CoCl_2_·6H_2_O, 98%), and PVP were obtained from Sigma-Aldrich. Sodium hydroxide pellets (NaOH, 98%) were purchased from Daejung Chemical & Metals Co. Ltd. Hydrazine monohydrate (N_2_H_4_·H_2_O, 80%) was obtained from Duksan Pure Chemicals. All chemicals were of analytical grade and were used without further purification.

To synthesize cNNH, an alcohol solution (25 mL) containing 0.1 M NiCl_2_·6H_2_O as a precursor solution was prepared in a 100 mL vial. Next, 6 mL of N_2_H_4_·H_2_O was added dropwise to the precursor solution. After vigorous stirring for 30 min, 10 mL of the mixed NiCl_2_·6H_2_O and N_2_H_4_·H_2_O solution was added dropwise to 20 mL of ethanol containing 1.4 g PVP40. Then, 20 mL of 0.1 M NaOH solution was added further. The prepared solution was transferred to a Teflon-lined stainless steel autoclave. The autoclave was subsequently heated in an electric oven at 160 °C for 3 h. After natural cooling, the black precipitate was collected by centrifugation at 5000 rpm for 10 min. The precipitates were washed several times with acetone, ethanol, and distilled water and eventually dried at room temperature. 

**Figure 1:**
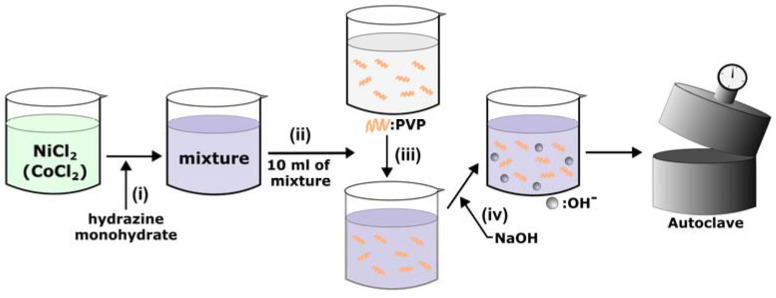
Schematic illustration of the synthesis of cNNH. (**i**) Insertion of the hydrazine monohydrate into the NiCl_2_ (additional CoCl_2_ in Co-doped cNNH) precursor, (**ii**) 10 mL extraction of a mixture, (**iii**) insertion of the mixture into the PVP precursor, and (**iv**) insertion of NaOH into the mixture.

In order to investigate the effect of each reagent on the nanostructure, the resultants with respect to the presence and absence of each reagent were observed. To synthesize Co-doped cNNH, 17.7 mg of CoCl_2_·6H_2_O was initially added to the NiCl_2_·6H_2_O precursor solution. A schematic of the synthesis is shown in Figure 1.

X-ray diffraction (XRD, PANalytical diffractometer) was used to identify the crystalline structures. The surface morphology was analyzed using field-emission scanning electron microscopy (FE-SEM, Hitachi SU8220). Transmission electron microscopy (TEM) and energy-dispersive X-ray spectroscopy (EDS) elemental mapping were performed using a JEOL JEM-2100F instrument at an accelerating voltage of 200 keV. The surface chemical states of the products were measured using X-ray photoelectron spectroscopy (XPS, Thermo Fisher NEXSA). Fourier-transform infrared (FT-IR, PerkinElmer Frontier) spectroscopy was used to characterize the functional groups on the surfaces of the samples. The electrochemical properties were evaluated by cyclic voltammetry (CV), galvanostatic charge–discharge (GCD), and electrochemical impedance spectroscopy (EIS) measurements using an Ivium Vertex. All the electrochemical properties of the samples were measured in a three-electrode system using Hg/HgO as the reference electrode, Pt mesh as the counter electrode, and 1.0 M KOH aqueous solution as the electrolyte. To fabricate the working electrode, Ni foam cleaned with ethanol and deionized water was inserted into a hydrothermal reactor for deposition on the surface of the Ni foam. The masses of the electroactive materials were approximately 3.2 mg. The loaded masses were estimated from the mass difference before and after the synthesis (OHAUS, EX125D). The specific capacitance was calculated from the GCD curves as follows: C_sp_ = (I × Δt)/(m × ΔV), where C_sp_ (F g^−1^) is the specific capacitance, I (A) is the discharge current, m (g) is the mass of the active material, Δt (s) is the total discharge time, and ΔV (V) is the potential drop during discharge. EIS measurements were performed by applying an AC voltage with an amplitude of 5 mV in the frequency range of 250 kHz to 0.01 Hz.

## 3. Results

### 3.1. Formation of Nanostructure

The schematic diagram in Figure 1 shows the synthetic process of cNNH. The cNNH synthesis process is listed sequentially from precursor preparation to hydrothermal synthesis. The addition of reagents at each step is specified, and the resultants with respect to the presence or absence of each reagent were also synthesized. Through these results, the effect of each reagent on nanostructure synthesis was investigated.

**Figure 2:**
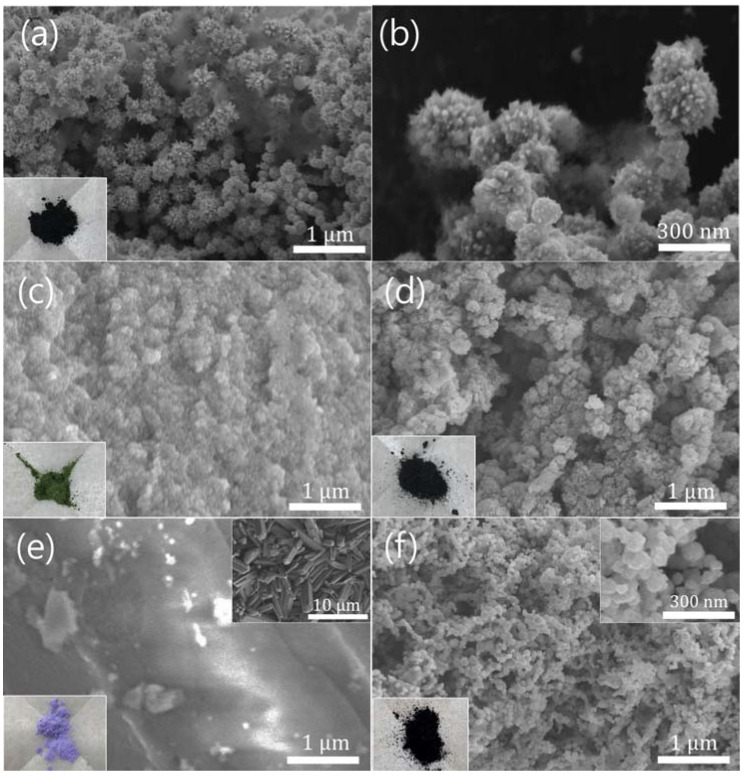
SEM images of (**a**) cNNH, (**b**) magnified image of cNNH, (**c**) sample without adding hydrazine, (**d**) sample without adding PVP, (**e**) sample without adding NaOH, and (**f**) Co-doped cNNH. The photograph of each sample is shown in the respective insets.

Figure 2 shows the SEM images of the prepared samples. The cNNH sample’s spherical surface was covered in nano-sized spikes, as shown in Figure 2a,b. The diameter of the particles was ~300 nm. The color of the cNNH was black, as shown in the inset in Figure 2a. The samples synthesized without hydrazine monohydrate consisted of small particles with diameters of ~50 nm, as shown in Figure 2c. The synthesized particles were olive in color, as shown in the inset in Figure 2c. In the samples synthesized without PVP, particles of a few nanometers in size agglomerated to form irregular powders, as shown in Figure 2d. PVP acts as a surfactant in the synthesis process. It selectively adsorbs on the facets of Ni crystals and, thus, kinetically controls the growth rates of the crystals [16]. Therefore, the spike shapes did not form. The color of the sample without PVP was black, as shown in the inset in Figure 2d. As shown in Figure 2e, the samples synthesized without NaOH consisted of large rods with a length of ~10 μm. The color of the precipitate was pale violet (inset in Figure 2e), thereby implying that the samples synthesized without NaOH did not form Ni through the ligand exchange reaction during the heating process. The morphology of the Co-doped cNNH particles, shown in Figure 2f, was roughly spherical with diameters of up to 100 nm. The doping with Co ions had a significant influence on the morphology and particle size of cNNH.

**Figure 3:**
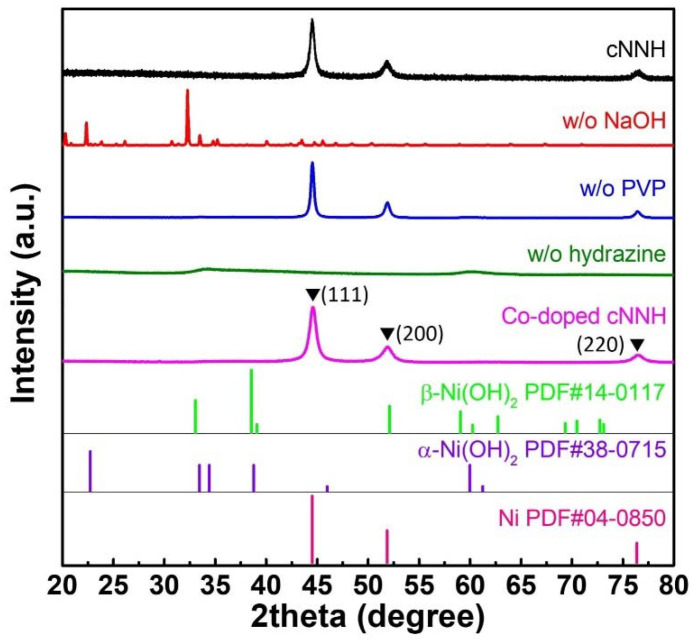
XRD patterns of cNNH, samples without NaOH, without PVP, without hydrazine, and with Co-doping.

As shown in Figure 3, the XRD pattern of cNNH exhibited three peaks at 44.5°, 51.8°, and 76.4°, which were indexed to the (111), (200), and (220) planes, respectively. These peaks match those of face-centered cubic Ni (JCPDS No. 04-0850). The low background intensity and relatively strong peaks indicate that the as-synthesized Ni particles were highly crystalline. The samples synthesized without hydrazine monohydrate exhibited two broad peaks, which were indexed to poorly crystalline Ni(OH)_2_. For the samples synthesized without PVP, diffraction peaks were observed at 44.5°, 51.8°, and 76.4°, which were indexed to the (111), (200), and (220) reflections of Ni, respectively. This pattern is the same as that of cNNH, which indicates that PVP can only affect the morphology, but not the crystallinity or phase of the materials. The diffraction patterns of the samples synthesized without NaOH showed a complicated pattern, suggesting that it was a mixed nickel hydrazine complex with a side product. The possible reactants are shown in Figure S1. The diffraction peaks of Co-doped cNNH were assigned to Ni. The peaks were slightly broader than those obtained for cNNH. This can be attributed to the different ionic radii of the doped Co ions in the crystalline Ni structure. Peaks for cobalt oxides, hydroxides, or other impurities were not detected, indicating that Co was well doped into cNNH.

**Figure 4:**
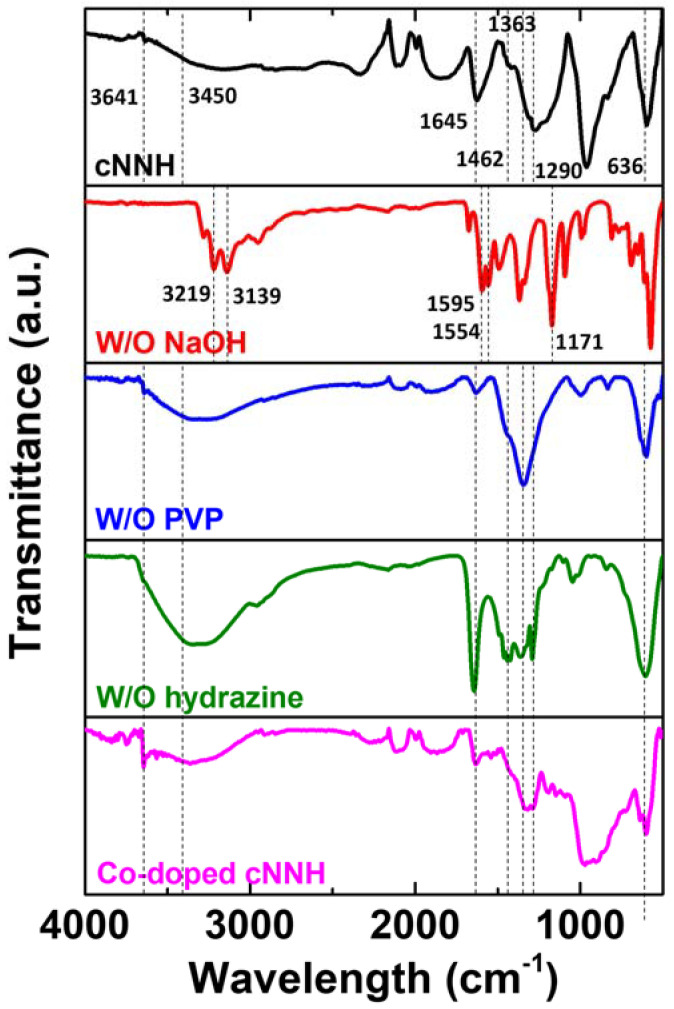
FTIR patterns of cNNH, samples without NaOH, without PVP, without hydrazine, and with Co-doping.

The as-prepared samples were analyzed using FTIR spectroscopy in the range of 400–4000 cm^−1^, and the results are shown in Figure 4. The broad peak at 3641 and 3450 cm^−1^ corresponds to the O–H stretching vibrations of the interlayer water molecules and hydrogen atoms bound to the OH^−1^ groups, which indicates the presence of hydroxyl ions in the samples. The peaks at approximately 1645 and 1462 cm^−1^ correspond to the bending vibration of H_2_O molecules, and the peak at 1363 cm^−1^ was attributed to the interlayer nitrate anion. The peak at 1290 cm^−1^ was attributed to C–N stretching. The band at approximately 636 cm^−1^ was assigned to the stretching vibrations of the Co–O and Ni–O bonds. These peaks were observed for the sample synthesized without hydrazine monohydrate, indicating that it was composed of Ni(OH)_2_. The bands of cNNH and Co-doped cNNH were similar to those of the sample synthesized without hydrazine solution, indicating the formation of Ni(OH)_2_. In contrast, the sample without the insertion of NaOH showed a different pattern than the other samples. Two peaks at 3219 and 3139 cm^−1^ were ascribed to anti-symmetric and symmetric stretching modes of N–H. The peaks at 1595 and 1554 cm^−1^ were assigned to the degenerate distortion, and the peak at 1171 cm^−1^ was the symmetric distortion [17]. These peaks were attributed to the presence of nickel hydrazine complexes. 

**Figure 5:**
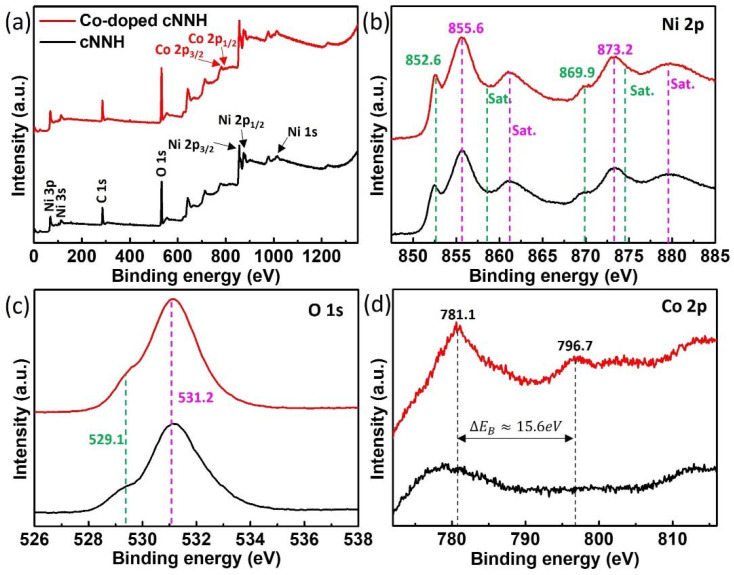
XPS spectra of cNNH and Co-doped cNNH. (**a**) Survey scan, (**b**) Ni 2p, (**c**) O 1s, and (**d**) Co 2p.

The chemical states of the elements bonded to cNNH and Co-doped cNNH were determined by XPS measurements of the Ni 2p, Co 2p, and O 1s core levels (Figure 5). The survey XPS spectra for cNNH and Co-doped cNNH are shown in Figure 5a, indicating the presence of Ni, Co, and O without impurities. Figure 5b shows a high-resolution Ni 2p spectrum composed of two chemical states. The peaks at approximately 852.6 and 869.9 eV with a spin–orbit separation of 17.3 eV indicate Ni^0^ corresponding to the metallic Ni. The peaks at binding energies of 855.6 and 873.2 eV with a spin–orbit separation of 17.6 eV can be assigned to Ni 2p_3/2_ and Ni 2p_1/2_ of Ni^2+^, respectively. These peaks were confirmed by the presence of Ni(OH)_2_ [7,10,18]. Figure 5c shows the O 1s core levels of cNNH and Co-doped cNNH. The strong peak at approximately 531.2 eV indicates O^2−^ of the hydroxide groups. The weak peak at approximately 529.1 eV was associated with the O atoms in Ni–O structures, which can be observed during oxidation in air [18,19]. The peaks of Ni 2p and O 1s clearly showed a difference from those of NiO, as shown in Figure S2.

The spectra of the Co 2p core level at 781.1 and 796.7 eV with a spin–energy separation of 15.6 eV correspond to the characteristic Co 2p_3/2_ and Co 2p_1/2_ peaks, respectively, as shown in Figure 5d, confirming the Co ion doping of cNNH [15,20].

**Figure 6:**
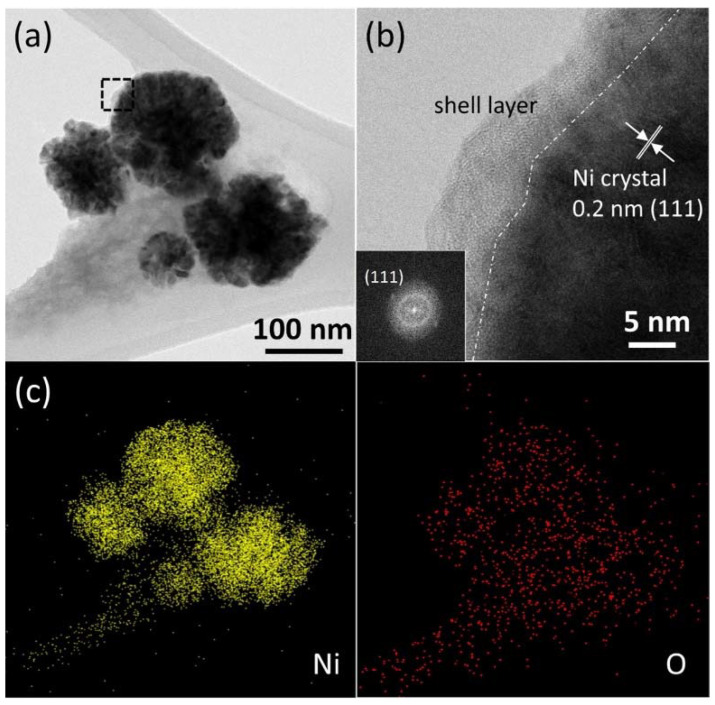
(**a**) Low- and (**b**) high-resolution TEM images of cNNH and (**c**) EDS mapping images of Ni and O (the inset of (**b**) shows the FFT image of crystalline Ni).

**Figure 7:**
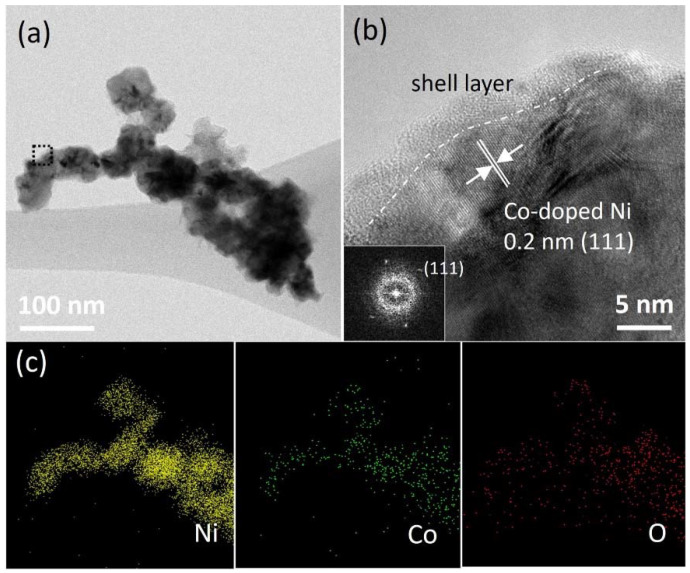
(**a**) Low- and (**b**) high-resolution TEM images of Co-doped cNNH and (**c**) EDS mapping images of Ni, Co, and O (the inset in (**b**) shows the FFT image).

The detailed morphology of cNNH and Co-doped cNNH was investigated via HR-TEM analysis. Figure 6 shows the TEM image and EDS data for cNNH. As shown in Figure 6a, cNNH was composed of thick inner particles and thin outer layers. The black dotted region in Figure 6a is enlarged and shown in Figure 6b, indicating that the low-crystalline layer can be distinguished from the crystalline core. The lattice spacing of the crystalline cNNH was measured to be 0.2 nm, corresponding to the d-spacing of the (111) lattice plane of Ni. The thickness of the shell layer was approximately 5 nm. The fast Fourier transform (FFT) pattern in the inset in Figure 6b exhibits a diffused ring and discernible diffraction spots, indicating the coexistence of an amorphous structure and crystalline Ni, respectively. The elemental mapping images of Ni and O in Figure 6c show that the species were homogeneously distributed in the particles. Figure 7 shows the TEM and EDS data of Co-doped cNNH, and Figure 7a shows that a thin layer covers the particles. The HR-TEM image of the dotted square region in Figure 7a shows the inner crystalline particle and the outer low-crystalline layer (Figure 7b). The measured lattice spacing corresponding to the d-spacing of the (111) lattice plane of Ni was 0.2 nm. The elemental mapping images of Ni, Co, and O (Figure 7c) show a uniform particle distribution. The inset in Figure 7b shows the corresponding FFT pattern of Co-doped cNNH. The diffraction spots identified in the pattern are consistent with the XRD results.

Crystalline cNNH and Co-doped cNNH were synthesized using the chemical reduction method. The main chemical reactions in the synthesis process can be expressed by the following equations [17,21,22,23]:

NiCl_2_ + 2N_2_H_4_ → [Ni(N_2_H_4_)_3_]Cl_2_ (1)

[Ni(N_2_H_4_)_3_]Cl_2_ + 2NaOH → Ni(OH)_2_ + 3N_2_H_4_ + NaCl (2)

2Ni(OH)_2_ + N_2_H_4_ → 2Ni(s) + N_2_ + 4H_2_O (3)

Hydrazine reduces NiCl_2_ to form a nickel–hydrazine complex, as shown in Equation (1). This complex exists as a mixture of Ni(N_2_H_4_)_2_Cl_2_, [Ni(N_2_H_4_)_3_]Cl_2_, and [Ni(N_2_H_4_)_6_]Cl_2_. The mixture’s composition depended on the [N_2_H_4_]/[Ni^2+^] molar ratio. In our synthesis, [Ni(N_2_H_4_)_3_]Cl_2_ was predominantly formed within the mixture, owing to the molar ratio of [N_2_H_4_]/[Ni^2+^] > 4.5. The color of the mixture was pale violet, and this was consistent with that reported in the literature [17,22]. When NaOH solution was added to the mixture, nickel hydroxide was formed by the ligand exchange of Cl^−^ ions by OH^−^ at temperatures above 60 °C, as shown in Equation (2). Next, nickel hydroxide was reduced by hydrazine liberated from the nickel hydrazine complex to form Ni, as indicated in Equation (3). As shown in the XRD, FT-IR, TEM, and XPS analyses, the nickel hydroxide residue covered the surface of the nickel particles. 

According to the observations of the materials, structural growth is suggested by the following process: the Ni nuclei are produced by the reduced Ni ion in Equation (3). With increasing temperature, these nuclei congregate to form Ni particles via Ostwald ripening. A difference in the growth rate exists in each facet of the particles due to PVP. Consequently, spikes grew on the surface of cNNH. After cooling, the remaining nickel hydroxides were deposited on Ni, as shown in the TEM images. In the case of adding Co ions, a lattice distortion was caused by the difference in the ionic radii of the Ni and Co ions. This could result in a decrease in the crystallinity and weaken the self-agglomeration of the nuclei.

### 3.2. Electrochemical Analysis of the cNNH and Co-Doped cNNH

**Figure 8:**
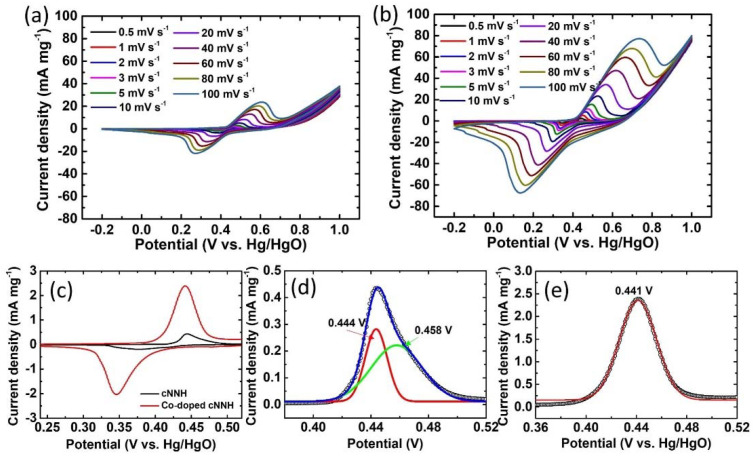
CV curves of (**a**) cNNH and (**b**) Co-doped cNNH at various scan rates; (**c**) redox peaks of cNNH and Co-doped cNNH at a scan rate of 0.5 mV s^−1^ on a magnified scale; fit oxidation peaks of (**d**) cNNH and (**e**) Co-doped cNNH.

Figure 8a,b show the CV curves of cNNH and Co-doped cNNH between −0.2 and 1.0 V at different scan rates. The two strong oxidation and reduction peaks were attributed to the reversible Faradaic reaction between Ni(II) and Ni(III) due to OH^−^ insertion/desertion. The oxidation and reduction peaks were nearly symmetric, indicating the excellent electrochemical reversibility of cNNH and Co-doped cNNH. The area under the CV curves of Co-doped cNNH was significantly greater than that of cNNH, indicating that Co-doped cNNH had a higher specific capacitance than cNNH. Figure 8c presents the redox peaks of cNNH and Co-doped cNNH at a scan rate of 0.5 mV s^−1^ on a magnified scale, indicating the difference between the two peaks. The cNNH peak shown in Figure 8d can be fit using the two peaks at approximately 0.444 and 0.458 V. The peak of Co-doped cNNH shown in Figure 8e can be fit using a peak at ~0.441 V. The CV peaks of nickel hydroxide can be attributed to two redox reactions, α-Ni(OH)_2_ ↔ γ-NiOOH and β-Ni(OH)_2_ ↔ β-NiOOH, because they exist in two different polymorphs. The oxidation of α-Ni(OH)_2_ to γ-NiOOH occurs at a lower potential than that of β-Ni(OH)_2_ to β-NiOOH [24,25]. This suggests that the peak at ~0.444 V is related to γ-NiOOH formation, whereas that at ~0.458 V is related to β-NiOOH formation. The β-Ni(OH)_2_ peak of cNNH can be attributed to the transformation due to instability in an alkaline aqueous environment. Co-doped cNNH was mainly attributed to the redox reaction, α-Ni(OH)_2_ ↔ γ-NiOOH. This indicates that Co-doping improves structural stability in alkaline media.

**Figure 9:**
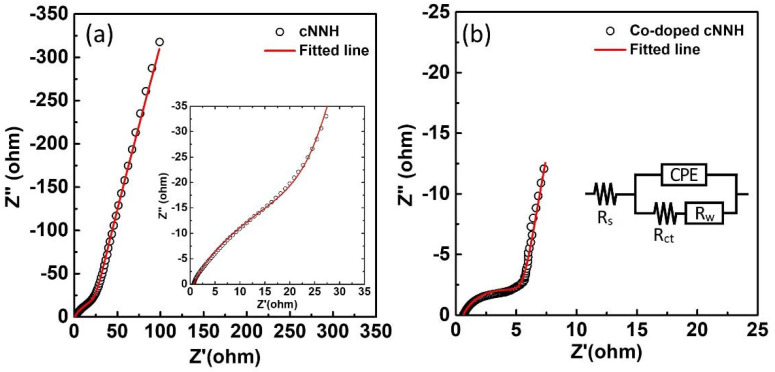
Nyquist plots of (**a**) cNNH and (**b**) Co-doped cNNH. The insets in (**a**) and (**b**) are the magnified high-frequency regions of cNNH and the equivalent circuit model, respectively.

Figure 9a,b show the Nyquist plots of cNNH and Co-doped cNNH, respectively. The Nyquist plots show compressed semicircles in the high-frequency region and inclined lines in the low-frequency region. The shapes were modeled using an equivalent circuit, as shown in the inset of Figure 9b. R_s_ is the intersection point on the Z’ axis in the high-frequency region, which corresponds to the combination of the resistance of the electrolyte and contact resistance. The R_s_ values of cNNH and Co-doped cNNH were approximately 0.67 and 0.63 Ω, respectively. R_ct_ obtained by estimating the diameter of the semicircle is the charge-transfer resistance, which is associated with the Faradaic reactions of the active materials. The semicircle observed for Co-doped cNNH had a significantly smaller diameter than that observed for cNNH, indicating that the R_ct_ of Co-doped cNNH (5.9 Ω) is lower than that of cNNH (36.7 Ω). The constant phase element (CPE) is the capacitance of the outermost surface of the electrode. A CPE was used instead of a capacitor owing to the inhomogeneity or roughness of the interface. R_w_ represents the inclined line in the low-frequency region and is the diffusive resistance of the electrolyte ions inside the nanomaterials. The results of the EIS analysis showed that the R_s_, R_ct_, and R_w_ of Co-doped cNNH were lower than those of cNNH.

**Figure 10:**
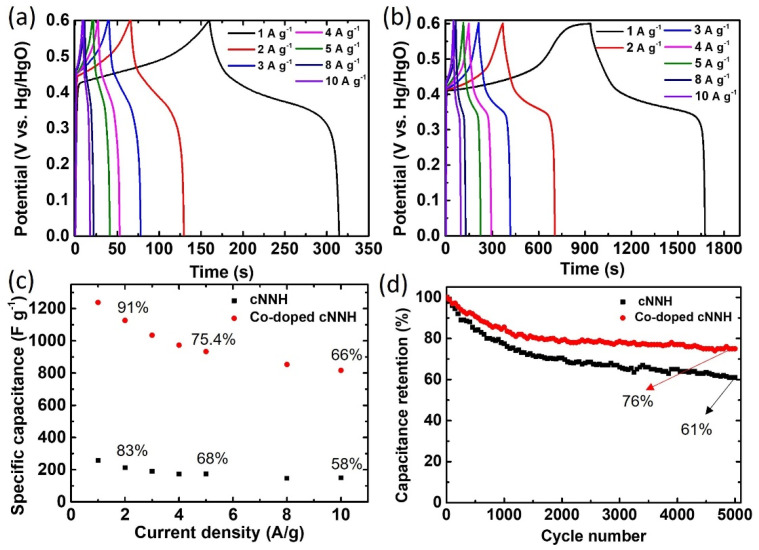
GCD curves of (**a**) cNNH and (**b**) Co-doped cNNH. (**c**) Calculated specific capacitance of cNNH and Co-doped cNNH at various current densities. (**d**) Cycle tests of cNNH and Co-doped cNNH at a current density of 3 A g^−1^.

Figure 10a,b show the GCD curves of cNNH and Co-doped cNNH in the range of 0–0.6 V at various current densities. The nonlinear discharge lines show that the Faradaic redox reaction occurred in the active materials, which is consistent with the CV curves. The calculated specific capacitances of cNNH were 213.3, 175, and 150 F g^−1^, and those of Co-doped cNNH were 1126.7, 933.3, and 816.7 F g^−1^ at current densities of 2, 5, and 10 A g^−1^, respectively (Figure 10c). The capacitance retention rates of cNNH and Co-doped cNNH were 83, 68, and 58% and 91, 75.4, and 66%, respectively. The Coulombic efficiency of Co-doped cNNH was above 98% at 3–10 A g^−1^, which implies good reversibility of the electrode. Figure 10d shows the cycle performances of cNNH and Co-doped cNNH at a current density of 3 A g^−1^ for 5000 cycles. The specific capacitance of Co-doped cNNH slowly decreased to 76% of the initial specific capacitance, thus demonstrating improved retention compared to cNNH. Electrochemical measurements showed that the electrochemical performance of cNNH was enhanced by doping it with the Co ions. In particular, doping with the Co ions decreased the size of the nanostructures, which increased the specific surface area and the accessibility of the electrolyte ions. Further, the Co ions enhanced the electrical conductivity of cNNH by increasing the number of free holes in the valence band [14]. This effect was confirmed by the significantly reduced resistance values evaluated through EIS analysis.

## 4. Conclusions

Based on a systematic investigation of the nanostructure synthesis mechanism, we successfully fabricated cNNH using a one-pot hydrothermal method. Nickel chloride reacted with hydrazine solution to form nickel hydrazine complexes. Nickel hydroxide was produced by a ligand exchange reaction between the nickel hydrazine complex and NaOH with increasing temperature. The hydrazine released from the ligand exchange reaction reduced nickel hydroxide, thereby forming small particles of Ni nuclei. During the growth of Ni particles by Ostwald ripening, the selectively adsorbed PVP on the facets of the particles controlled the growth rate. The obtained nanostructures consisted of a Ni core embedded in a thin hydroxide layer of a few nanometers. Co-doping significantly affected the growth of Ni particles during the agglomeration process by Ostwald ripening at high temperatures. The electrochemical performance tests of Co-doped cNNH resulted in a specific capacitance of 1238 F g^−1^ at 1 A g^−1^, 66% retention at 10 A g^−1^, and reduced resistance values obtained via EIS measurements, thus demonstrating the superior performance of Co-doped cNNH compared to cNNH. Therefore, this synthesis procedure provides prospects for nickel/nickel hydroxide materials for application in supercapacitors in a simple and low-cost fashion.

## Data Availability

All the data used in this study are already included in the manuscript.

## Supplementary Materials

The following supporting information can be downloaded at: https://www.mdpi.com/article/10.3390/ma16010380/s1.
