# Supplementary material for: One-Pot Synthesis of Nanostructured Ni@Ni(OH)2 and Co-Doped Ni@Ni(OH)2 via Chemical Reduction Method for Supercapacitor Applications
Source: Materials (Basel). 2022 Dec 30;16(1):380. doi: 10.3390/ma16010380 (PMC9822482; doi:10.3390/ma16010380)
Supplement: Supplementary file 1 [file materials-16-00380-s001.zip › materials-2087880-supplementary.pdf]

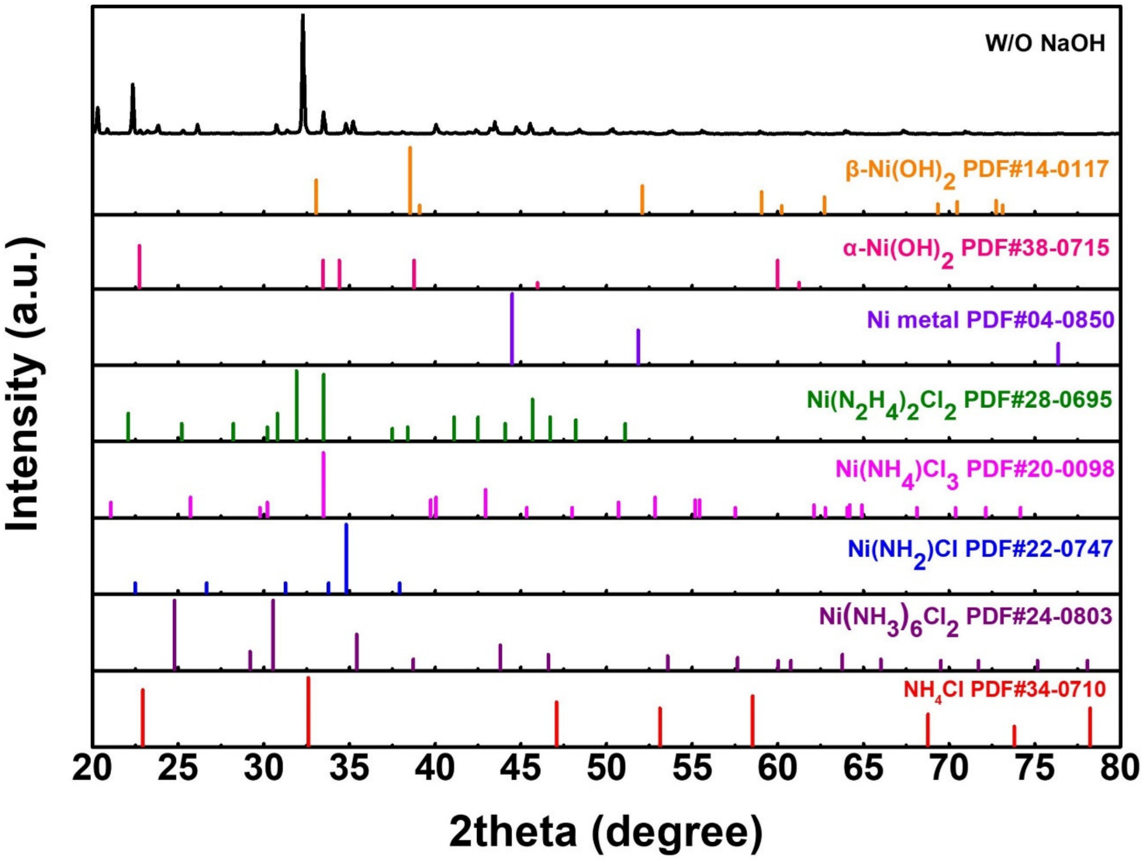

Figure S1. XRD patterns of the sample without NaOH and the potential candidates. The side products are formed when the nickel complexes are heated in the absence of NaOH.

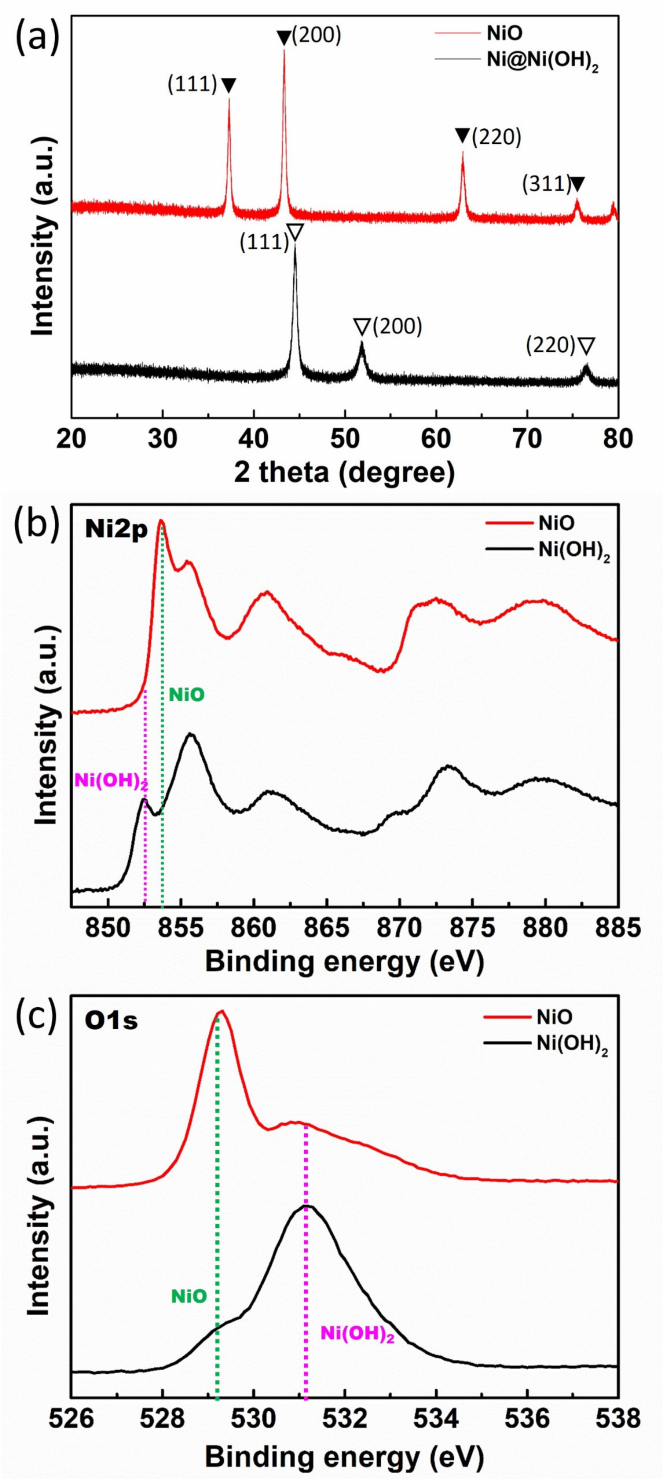

Figure S2. (**a**) XRD patterns of the cNNH and NiO. XPS spectra of cNNH and NiO. (**b**) Ni 2p, (**c**) O 1s, The NiO was obtained by the annealing of cNNH at 500 °C for 6 h. The XRD pattern of annealed cNNH could be indexed to the NiO phase (JCPDS # 47-1049).
